# bta-miR-23a Regulates the Myogenic Differentiation of Fetal Bovine Skeletal Muscle-Derived Progenitor Cells by Targeting *MDFIC* Gene

**DOI:** 10.3390/genes11101232

**Published:** 2020-10-20

**Authors:** Xin Hu, Yishen Xing, Ling Ren, Yahui Wang, Qian Li, Qiyuan Yang, Min Du, Lingyang Xu, Luc Willems, Junya Li, Lupei Zhang

**Affiliations:** 1 Institute of Animal Sciences, Chinese Academy of Agricultural Sciences, Beijing 100193, China; huxin19890803@163.com (X.H.); yishen_xing@163.com (Y.X.); renling5454@163.com (L.R.); wang1434243198@163.com (Y.W.); lq798711247@163.com (Q.L.); xulingyang@caas.cn (L.X.); lijunya@caas.cn (J.L.); 2 Molecular and Cellular Biology, Gembloux Agro-Bio Tech, University of Liège, 5030 Gembloux, Belgium; luc.willems@uliege.be; 3 Department of Molecular, Cell and Cancer Biology, University of Massachusetts Medical School, Worcester, MA 01655, USA; qiyuan.yang@umassmed.edu; 4 Washington Center for Muscle Biology and Department of Animal Sciences, Washington State University, Pullman, WA 99164, USA; min.du@wsu.edu

**Keywords:** bta-miR-23a, fetal muscle development, *MDFIC*

## Abstract

miR-23a, a member of the miR-23a/24-2/27a cluster, has been demonstrated to play pivotal roles in many cellular activities. However, the mechanisms of how bta-miR-23a controls the myogenic differentiation (MD) of PDGFRα^−^ bovine progenitor cells (bPCs) remain poorly understood. In the present work, bta-miR-23a expression was increased during the MD of ^PDGFRα−^ bPCs. Moreover, bta-miR-23a overexpression significantly promoted the MD of ^PDGFRα−^ bPCs. Luciferase reporter assays showed that the 3’-UTR region of *MDFIC* (MyoD family inhibitor domain containing) could be a promising target of bta-miR-23a, which resulted in its post-transcriptional down-regulation. Additionally, the knockdown of *MDFIC* by siRNA facilitated the MD of ^PDGFRα−^ bPCs, while the overexpression of *MDFIC* inhibited the activating effect of bta-miR-23a during MD. Of note, *MDFIC* might function through the interaction between *MyoG* transcription factor and *MEF2C* promoter. This study reveals that bta-miR-23a can promote the MD of ^PDGFRα−^ bPCs through post-transcriptional downregulation of *MDFIC*.

## 1. Introduction

The development of skeletal muscles can occur as early as the embryonic period [1]. During the embryonic process, the first myogenesis wave involves the formation of primary myofibers, while the second myogenesis wave facilitates the production of secondary myofibers that account for the majority of skeletal muscle fibers. Considering that there is no available information on the increased number of muscle fibers following birth, the embryo-fetal stage can be considered a great determinant of skeletal muscle development [2,3,4]. Differentiation of skeletal muscles involves complex and multilevel processes, and such complex differentiation processes are orchestrated by a variety of myogenic regulatory factors, such as myogenic differentiation (*MyoD*), myogenin (*MyoG*), myogenic factor 5 (*Myf5*), and myogenic factor 6 (*Myf6*). *MyoD* and *Myf5* are both transcription factors for initiating myogenic commitment [5]. *Myf6* is involved in the processes of differentiation and maturation of myotubes during embryogenesis [6]. *MyoG* is essential for the terminal differentiation of skeletal myoblasts to form myotubes [7]. There are four types of myosin heavy-chain (MyHC) isoforms in skeletal muscles, including type I, IIa, IIx, and IIb. These isoforms are encoded by *MyHC* 7, *MyHC 2*, *MyHC 1,* and *MyHC 4* genes, respectively. Extensive research shows that different MyHC isoforms have considerable effects on meat quality. Type I muscle fibers are thinner, and a high type I muscle fibers ratio is beneficial for improving meat tenderness [8]. Furthermore, other research also found that muscle with a higher type I muscle fiber ratio contained more intramuscular fat. The cross-sectional region of type II muscle fibers is also greater, and an elevated type II fiber ratio can lead to increases in muscle mass and animal weight [9].

MicroRNA is an endogenous, small non-coding RNA of ~22 nucleotides, which negatively modulates gene expression levels by repressing translation and degradation of mRNA through binding to 3’-UTR (3’-untranslated region) [10,11]. Emerging evidence has indicated that a number of miRNAs can play vital roles during muscle development. For instance, miR-1, -133, and -206 have been recognized as muscle-specific miRNAs that modulate the function of skeletal muscles. miR-1 and -206 modulate the satellite cell MD of bovine skeletal muscles by downregulating histone deacetylase 4 *(HDAC4)* and paired box 7 *(Pax7)* expression [12]. According to the findings of miRNA microarray and sequencing analysis, miR-1, −133 and −206 are remarkably upregulated during the MD of bovine satellite cells [13,14]. Moreover, non-muscle-specific miRNAs can also regulate the development of bovine skeletal muscles. In Qinchuan cattle, miR-101-1 is enriched in skeletal muscles, and negatively regulates the differentiation of C_2_C_12_ cells [15]. In addition, miR-27b plays prominent roles in the development and hypertrophy of bovine skeletal muscles [16]. In bovine myoblast cells, miR-483 suppresses bovine myoblast cell growth and differentiation by negatively regulating IGF1/PI3K/AKT pathway [17]. Some research showed that miR-23a suppressed the translation of *MuRF1* and *MAFbx/atrogin-1* in a 3’-UTR-dependent fashion, and its forced expression in myofibers and myotubes could prevent muscle atrophy [18]. In addition to that, miRNA-23a has been reported as a critical regulator in colorectal cancer [19,20], lung cancer [21], pancreatic cancer [22], gastric cancer [23] and hepatocellular carcinoma [24]. Our previous work also found that miR-23a was highly upregulated during MD. Nevertheless, the impact of miR-23a on the MD of fetal bovine skeletal muscles has yet to be determined.

*MDFIC* (MyoD family inhibitor domain containing) belongs to a small family of proteins encompassing the cysteine-rich C-terminal domain [25,26]. Whilst, *MyoD* family inhibitor isoform 1 (*MDFI*, or named as I-mf or I-mfa) is another member of this family. The cysteine-rich C-terminal domain of *MDFIC* contains 81 amino acids, which is 77% identical to that of *MDFI*. Approximately 26 and 24 cysteine residues are found in the C-terminal domains of *MDFI* and *MDFIC*, respectively. *MDFI* restricts the transactivation functions of *MyoD* family members and prevents MD through binding the basic helix-loop-helix (bHLH) domain of MyoD with cysteine-rich domain [27]. *MDFI* regulates myogenesis by preventing DNA binding and nuclear localization of *MyoD* and bHLH proteins [28]. Although *MDFIC* shares relatively high identity with *MDFI* based on their amino acid sequences, the functional role of *MDFIC* in myogenesis has not been addressed. A genome-wide association study on pig has identified *MDFIC* as a candidate gene for determining piglet birth weight [29].

Hence, the current research aimed to identify the roles of bta-miR-23a in the differentiation of fetal bovine myogenic cells, and its relationship with *MDFIC*. The findings demonstrated that bta-miR-23a could promote myoblast differentiation via targeting *MDFIC*. Overall, this study provides new evidence supporting the potential application of bta-miR-23a for preventing muscle atrophy.

## 2. Materials and Methods 

### 2.1. Animal Ethics Statement

The experiment was carried out in strict accordance with the guidelines of the Regulations for the Administration of Affairs Concerning Experimental Animals (Ministry of Science and Technology, China). The ethical approval was obtained from the Institutional Animal Ethics Committee, Chinese Academy of Agricultural Sciences, China (No. IAS2019-48). Pregnant cattle were raised by the Aokesi Agricultural Technology Co., Ltd. (Inner Mongolia, China). Every effort was made to reduce the suffering of pregnant cattle.

### 2.2. Cell Culture and Treatment

bPCs were enzymatically isolated from the bovine fetus-derived longissimus dorsi tissues at 90–120 days according to previously reported method [30]. Longissimus dorsi was cut into smaller pieces with scissors, and then digested with 0.1% type-IV collagenase (Sigma-Aldrich, St. Louis, MO, USA) at 37 °C. Digestions were terminated by low-glucose DMEM (Gibco, Grand Island, NY, USA) containing 10% fetal bovine serum (FBS; Gibco) after 1 hour. After filtering through a 40-μm nylon mesh, the filtered cells were collected through centrifugation, and then resuspended in ice-cold phosphate-buffered saline (PBS) buffer with 0.5% bovine serum albumin (BSA) and 2 mM EDTA. The cells were then pretreated with anti-platelet-derived growth factor receptor α (PDGFRα) antibody for 30 m at 4 °C. Washing and resuspending with PBS first and incubating with Anti-Rabbit IgG MicroBeads (Miltenyi Biotec, Bergisch Gladbach, Germany) for 15 minutes at 4 °C. After harvesting and resuspending in buffer, the cells were subjected to magnetic separation with the Mini-MACS system (Miltenyi Biotec). The obtained cells were then cultured in DMEM containing 10% FBS, followed by incubation at 37 °C (5% CO2 atmosphere). Upon reaching 70–80% confluency, the cells were detached using trypsin-EDTA (0.25%; Gibco) and subjected to passaging. When achieving 100% confluency [day 0 (D0)], the cells were differentiated to day 2 (D2), day 3(D3), and day 5 (D5) in DMEM containing 5% horse serum (Gibco).

HEK293 (human embryonic kidney 293) cells were cultured in DMEM medium containiing 10% FBS, 100 U/mL penicillin and 100 μg/mL streptomycin, followed by incubation at 37 °C (5% CO_2_ atmosphere).

### 2.3. RNA Extration and Real-Time PCR Assay

TRIzol reagent (Invitrogen, Carlsbad, CA, USA) was utilized to isolate total RNA from the culturing cells. The yield and quality of RNA were evaluated by an Implen NanoPhotometer N50 (Munich, Germany) and 1% agarose gel electrophoresis, respectively. The results showed three clear rRNA bands of 28S, 18S, and 5S. The ratio of the optical densities measured at 260 and 280 nm were >1.9 for all RNA samples. The conversion of 500 ng total RNA to cDNA was initiated with PrimeScript RT Master Mix (TaKaRa Bio, Kusatsu, Japan), while the reverse transcription reaction for miRNA was conducted with miRcute miRNA First-Stand cDNA Synthesis Kit (TIANGEN, Beijing, China). The real-time PCR reactions were carried out in triplicate on a QuantStudio™ 7 Flex RT-PCR System using KAPA SYBR^®^ FAST qPCR Kit (KAPABiosystems, Wilmington, MA, USA) and miRcute miRNA qPCR Kit (TIANGEN). PCR primers for amplification of mRNA and miRNA-specific add the poly(A)-tail primers were designed by Primer Premier 5.0 and synthesized by ShengGong. All experiments were conducted as per the manufacturer’s recommended protocols. The primer sequences for mRNA and miRNA detection are listed in Table 1.

### 2.4. Immunofluorescence Assay

First, the cells in 12-well plate were fixed in 4% paraformaldehyde for 15 minutes and rinsed 3 times for 5 minutes each in PBS. Then, the cells were permeabilized with 0.1% Triton X-100 for 10 minutes, followed by blocking with 1% BSA (Beyotime, Shanghai, China) for 30 minutes. Next, the cells were incubated with anti-MyHC antibody (1:100; Developmental Studies Hybridoma Bank [DSHB], Iowa City, IA, USA) at 4 °C overnight, and then with FITC-labeled Goat Anti-Mouse IgG (H + L; 1:1000; Beyotime) at room temperature for 1 hour. After DAPI (Sigma-Aldrich) staining for 5 minutes, the cell nuclei were examined using a TCS SP8 confocal microscope (Leica, Wetzlar, Germany).

### 2.5. Western Blotting

Total protein was isolated using the proteinase inhibitor-containing lysis buffer. Equivalent amounts of protein were separated on a 4–12% SurePAGE gel (GenScript, Nanjing, China). After electrophoresis, the separated proteins were placed onto a nitrocellulose membrane (Pall, Mexico), and then inhibited with 5% (w/v) skimmed milk. The blocked membrane was incubated overnight at 4°C with MDFIC (1:500; Biorbyt, Cambridge, UK), MyoG (1:1000; Santa Cruz, CA, USA), MyHC (1:50; DSHB, USA) or β-tubulin (1:2000; Proteintech, Chicago, IL, USA). After rinsing 3 times in Tris-buffered saline/Tween, the membranes were incubated with the corresponding HRP-labeled Goat Anti-Rabbit IgG (1:1000; Beyotime) or HRP-labeled Goat Anti-Mouse IgG (1:1000; Beyotime) for 1 hour at room temperature. The protein blots were visualized using the Enhanced Chemiluminescent Reagent (Beyotime). 

### 2.6. RNA Oligonucleotides, Plasmids Construction and Cell Transfection

The design and synthesis of bta-miR-23a mimic, mimic negative control (NC), small interfering RNAs (siRNAs) for *MDFIC* knockdown and non-targeting siRNA NC were performed by RiboBio (Guangzhou, China). The sequence of si-MDFIC was GAATCGAAGACTTTCAGCA. The 3’-UTR region of *MDFIC* encompassing bta-miR-23a binding site was amplified and cloned into the XhoI/NotI restriction sites of psi-CHECK2 vectors (Promega, Madison, WI, USA). Then, site-directed mutagenesis was carried out using the Fast Site-Directed Mutagenesis Kit (TIANGEN). 

To obtain the *MDFIC* overexpression plasmid, *MDFIC* open reading frame sequence was amplified and cloned into pBI-CMV3 vector (Clontech, Mountain View, CA, USA) using ClonExpress MultiS One Step Cloning Kit (Vazyme, Nanjing, China). *MEF2C* promoter sequence was taken out and cloned into pGL3-basic vector by using ClonExpress MultiS One Step Cloning Kit (Vazyme). The primer sequences used for plasmid construction and mutagenesis are listed in Table 2.

Cell transfection was conducted with Lipofectamine RNAiMAX reagent (Invitrogen), along with bta-miR-23a mimic, NC, si-MDFIC and si-NC. Plasmid transfection was carried out with Lipofectamine 3000 (Invitrogen). All assays were conducted as per the manufacturer’s protocols.

### 2.7. Bioinformatic Analyses

TargetScanHuman 7.2 (http://www.targetscan.org/vert_72/) was employed for the prediction of the target genes of bta-miR-23a. The predicted target genes with a context ++ score lower than −0.3 were selected for Gene Ontology (GO) and Kyoto Encyclopedia of Genes and Genomes (KEGG) pathway enrichment analyses were performed using the web-based DAVID bioinformatics resources 6.8 (https://david.ncifcrf.gov/home.jsp) and KOBAS 3.0 (http://kobas.cbi.pku.edu.cn/kobas3). All GO terms and KEGG pathways with *p*-value below 0.05 and a minimum of 5 genes were chosen for subsequent analyses.

### 2.8. Dual-Luciferase Reporter Assay

For luciferase detection, the HEK293 cell line was co-transfected with bta-miR-23a mimic or NC and the wild-type or mutant plasmid in a 96-well plate using the Lipofectamine 3000 reagent (Invitrogen). ^PDGFRα−^ bovine progenitor cells seeded in a 48-well plate and co-transfected with plasmid of *MEF2C* promoter sequence with OV-MDFIC or si-MDFIC. After transfection for 48 h, the activities of luciferase reporter (LR) were evaluated using a Dual-LR Assay System (Promega).

### 2.9. Statistical Analysis

The comparative (^2-ddCt^) method was used to present relative mRNA expression level. The protein level was normalized to β-tubulin. Differentiation index was the percentage of nuclei in MyHC–positive cells. The relative luciferase activity was monitored by renilla/firefly. The results of the qPCR, differentiation index and luciferase reporter assays from the cell culture experiments are presented as independent biological replicates, while the tissue qPCR analyses were analyzed using three individual biological replicates. The data were plotted by GraphPad Prism ver. 6.0 (GraphPad Software Inc., San Diego, CA, USA) and represented as the mean (± standard deviation (SD)). The difference was considered as statistically significant when the *p*-value < 0.05 (*) or *p*-value < 0.01 (**) 

## 3. Results

### 3.1. bta-miR-23a Expression Profile and Its Targets Analysis 

**Figure 1:**
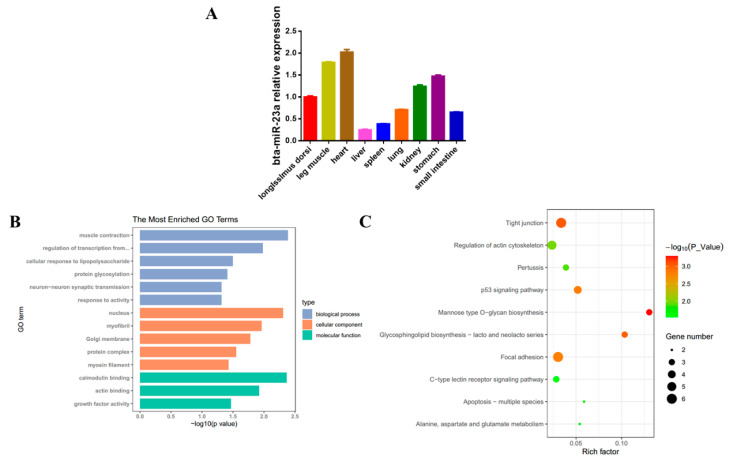
bta-miR-23a expression profile and its targets analysis. (**A**) Tissue expression of bta-miR-23a examined by qPCR in fetal tissues. The fold change of bta-miR-23a was relative to bta-miR-23a expression of longissimus dorsi. (**B**) GO term enrichment of target genes for bta-miR-23a. (**C**) KEGG pathway analysis of target genes for bta-miR-23a. Results are representative of the mean (± SD) of three independent analyses.

In our previous work [31], we observed that bta-miR-23a was highly upregulated during MD. The expression levels of bta-miR-23a were then detected in different tissues, such as heart, kidney, leg muscle, liver, longissimus dorsi, lung, small intestine, spleen and stomach tissues derived from fetal bovine, and it was found that bta-miR-23a had ubiquitous expression patterns in different tissue types (Figure 1A). GO term enrichment was further conducted on bta-miR-23a target genes, and the results demonstrated that bta-miR-23a was involved in muscle contraction, myofibril, myosin filament, growth factor activity and nucleus (Figure 1B). Moreover, KEGG enrichment analysis demonstrated that bta-miR-23a might be related to tight junction, focal adhesion, glycosphingolipid biosynthesis, p53 signaling pathway, and the regulation of actin cytoskeleton (Figure 1C). Altogether, these data imply that bta-miR-23a is a potential regulator in myogenesis.

### 3.2. bta-miR-23a Is Upregulated During MD of ^PDGFRα−^ bPCs

**Figure 2:**
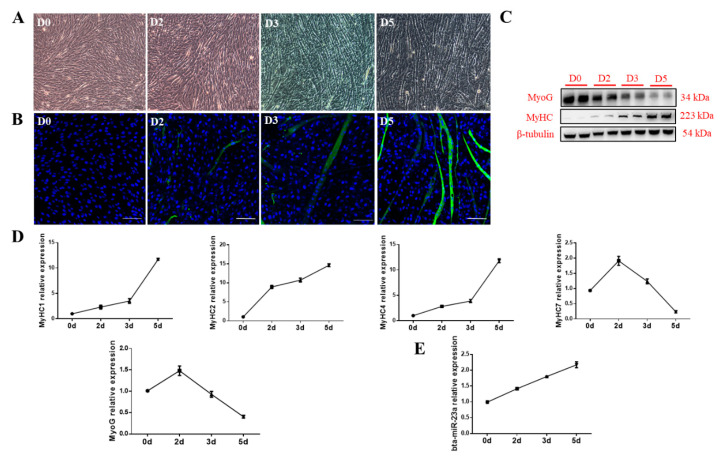
bta-miR-23a expression increased during myogenic differentiation (MD) of ^PDGFRα−^ bPCs. (**A**) Morphological features of ^PDGFRα−^ bPCs at Day 0 (D0), 2 (D2), 3 (D3) and 5 (D5) after MD. Scale bar represents 100 µm. (**B**) MyHC positive cells (green) at D0, D2, D3 and D5 after MD were examined by fluorescence microscopy. Scale bar represents 100 µm. The protein (**C**) and mRNA (**D**) levels of MyoG and MyHC. β-tubulin was used as the reference gene. (**E**) Relative expression of bta-miR-23a at D0, D2, D3 and D5 after MD. Results are representative of the mean (± SD) of three independent analyses.

To explore the potential role of bta-miR-23a, ^PDGFRα−^ bPCs were isolated from fetal bovine skeletal muscles. It was observed that ^PDGFRα−^ bPCs could form myotubes after 2 days of myogenic induction (Figure 2A,B). During MD, MyoG expression was downregulated, while MyHC expression was increased (Figure 2C). Moreover, the expression of four *MyHC* genes was evaluated during MD. It was found that the expression levels of *MyHC-1*, *-2*, and *-4* were upregulated during MD, while *MyHC-7* expression level was increased during first two days and then decreased afterwards (Figure 2D). Besides, the expression trend of bta-miR-23a was shown to be increased during MD (Figure 2E). These results suggest that our in vitro MD model is reliable for studying the differentiation of ^PDGFRα−^ bPCs in fetal bovine skeletal muscle.

### 3.3. bta-miR-23a Promotes the MD of ^PDGFRα−^ bPCs

**Figure 3:**
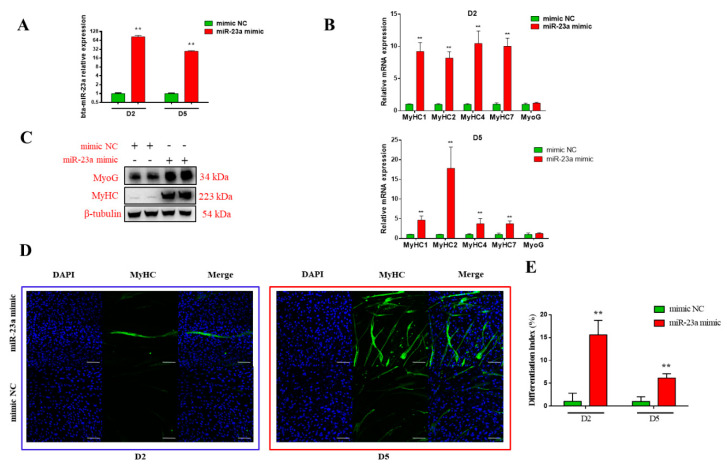
bta-miR-23a promoted MD of ^PDGFRα−^ bPCs. (**A**) The mRNA levels of bta-miR-23a in ^PDGFRα−^ bPCs after 48-h transfection with bta-miR-23a mimics or NC mimics. (**B**) The mRNA expression of *MyoG* and *MyHCs* in ^PDGFRα−^ bPCs at D2 and D5 after MD. (**C**) The protein levels of MyoG and MyHC in ^PDGFRα−^ bPCs at 96 h after MD. β-tubulin was used as the reference gene. (**D**) MyHC (green) positive cells at D2 and D5 after MD. Scale bar represents 100 µm. (**E**) Differentiation index of myoblast after induction for D2 and D5. Results are representative of the mean (± SD) of three independent analyses. ** *p* < 0.01.

To verify the biological effects of bta-miR-23a on MD, we introduced bta-miR-23a mimic and NC into ^PDGFRα−^ bPCs on Day 0. The activities of bta-miR-23a in mimic group were 85 and 27 times higher compared to NC group (*p* < 0.01) on Day 2 and 5 after transfection, respectively (Figure 3A). Further, transfection of bta-miR-23a mimic or NC into the cells was carried out at Day 0 after MD. The mRNA levels of *MyHC1*, *MyHC2*, *MyHC4*, and *MyHC7* were noticeably upregulated on Day 2 and 5 (Figure 3B). Similarly, bta-miR-23a overexpression increased the protein levels of MyoG and MyHC at Day 5 after MD (Figure 3C). Based on the immunofluorescence staining of myosin heavy chain, a high number of cells underwent differentiation and formed myotubes in mimic group compared to NC group on Day 2 and 5 (Figure 3D,E). Taken together, bta-miR-23a is shown to promote the MD of ^PDGFRα−^ bPCs.

### 3.4. MDFIC Is A Promising Target Gene of bta-miR-23a

**Figure 4:**
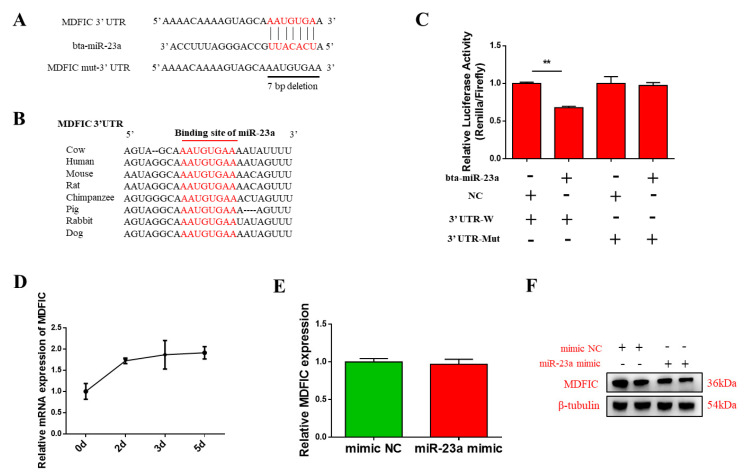
*MDFIC* gene is directly targeted by bta-miR-23a. (**A**) The predicated binding sites of bta-miR-23a in the 3′-UTR of *MDFIC*. Deletion of the seed region (in red) in the mutant 3′-UTR reporter. (**B**) The conservation of mature bta-miR-23a binding site in eight different species. (**C**) The relative luciferase activities of HEK293T cells co-transfected with the MDFIC 3′-UTR mutant or wild-type dual-luciferase reporter and bta-miR-23a mimic or the mimic NC at 48 h. (**D**) Expression pattern of *MDFIC* during MD. The mRNA (**E**) and protein (**F**) levels of MDFIC in ^PDGFRα−^ bPCs transfected with bta-miR-23a mimics or negative control at 48 h. β-tubulin was used as the reference gene. Results are representative of the mean (± SD) of three independent analyses. ** *p* < 0.01.

To elucidate the underlying mechanisms of bta-miR-23a-regulated gene expression, its target genes were predicated using the TargetScan databases. The findings indicated that *MDFIC* could be a promising target of bta-miR-23a (Figure 4A,B). To verify whether *MDFIC* is the direct target gene of bta-miR-23a, 2 LR plasmids consisting of either the wild-type or mutant 3′ -UTR of *MDFIC* gene were constructed (Figure 4A). Co-transfection of bta-miR-23a mimic or NC into HEK293T cells was carried out. Notably, bta-miR-23a markedly decreased the activity of wild-type *MDFIC* LR in comparison with those of negative control, but no significant changes were noted for the mutant LR (Figure 4C). These findings indicate that bta-miR-23a can indeed directly target the 3′-UTR of *MDFIC*. Moreover, we detected the expression of MDFIC during MD (Figure 4D). To examine the validity of the putative target, bta-miR-23a mimic or NC was transfected into ^PDGFRα−^ bPCs. RT-qPCR data revealed that the transcriptional levels of *MDFIC* were not obviously different between the mimic and NC groups (Figure 4E). Interestingly, Western blot analysis indicated that the translational levels of MDFIC were remarkably downregulated by bta-miR-23a mimic compared to NC group (Figure 4F). Thus, we concluded that bta-miR-23a can directly target the 3’-UTR of *MDFIC* to suppress its mRNA translation in ^PDGFRα−^ bPCs.

### 3.5. Knockdown of MDFIC Promotes MD

**Figure 5:**
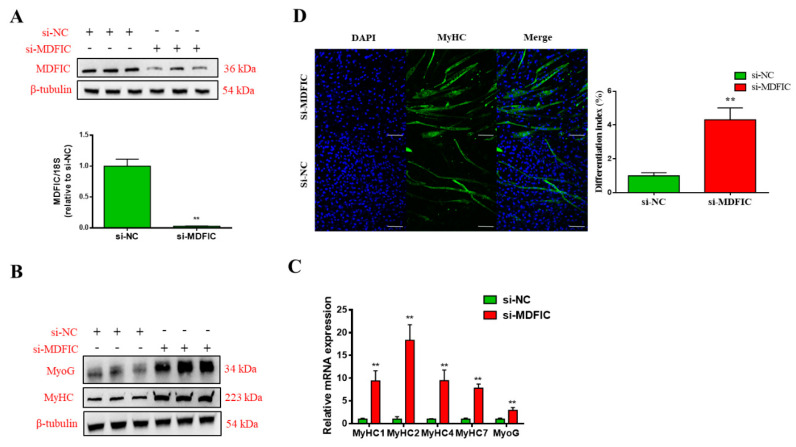
Knockdown of *MDFIC* promotes the MD of ^PDGFRα−^ bPCs. (**A**) The mRNA and protein expression level of MDFIC in ^PDGFRα−^ bPCs transfected with si-MDFIC or si-NC. β-tubulin was used as the reference gene. The protein (**B**) and mRNA (**C**) expression levels of MyoG and MHC in ^PDGFRα−^ bPCs transfected with si-MDFIC or si-NC at D5 after MD. β-tubulin was used as the reference gene. (**D**) MyHC positive cells (green) at D5 of MD after si-MDFIC or si-NC transfection. Scale bar represents 100 µm. Differentiation index of myoblast after induction for D5. Results are representative of the mean (± SD) of three independent analyses. ** *p* < 0.01.

To explore the roles of *MDFIC* during MD, ^PDGFRα−^ bPCs were transfected with siRNA against *MDFIC* or NC. siRNA significantly diminished *MDFIC* mRNA and protein expression compared to NC group (Figure 5A). The transcriptional and translational levels of MyoG and MyHC were obviously higher in siRNA group than those in NC group at Day 5 after MD (Figure 5B,C). Immunofluorescence staining revealed that the knockdown of *MDFIC* notably enhanced the process of MD (Figure 5D). Taken altogether, our findings indicate that the inhibition of *MDFIC* can promote MD and myogenesis-specific gene expression.

### 3.6. Overexpression of MDFIC Rescues the bta-miR-23a-Induced Effects

**Figure 6:**
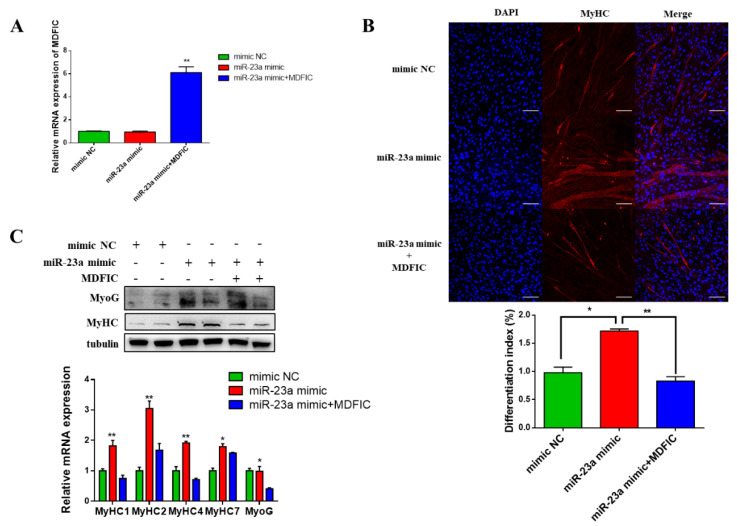
Overexpression of *MDFIC* rescues the miR-23a-induced effects. (**A**) The mRNA levels of *MDFIC* in ^PDGFRα−^ bPCs transfected with mimic NC, miR-23a mimic or miR-23a mimic+MDFIC. (**B**) MyHC (red) positive cells of MD after transfection with mimic NC, miR-23a mimic or miR-23a mimic + MDFIC. Scale bar represents 100 µm. Differentiation index of myoblast after transfection with mimic NC, miR-23a mimic or miR-23a mimic + MDFIC. (**C**) The protein and mRNA expression levels of MyHC and MyoG in ^PDGFRα−^ bPCs transfected with mimic NC, miR-23a mimic or miR-23a mimic+MDFIC. β-tubulin was used as the reference gene. Results are representative of the mean (± SD) of three independent analyses. * *p* < 0.05, ** *p* < 0.01.

To further determine the regulation effect between bta-miR-23a and *MDFIC*, we performed rescue experiments in myoblast differentiation. The mRNA level of *MDFIC* was significantly upregulated after *MDFIC* overexpression (Figure 6A). Meanwhile, the results showed that overexpression of *MDFIC* weakened the bta-miR-23a-induced effects, thus reducing the formation of myotubes, mRNA and protein levels of MyHC1, MyHC2, MyHC4, MyHC7 and MyoG (Figure 6B,C). All these results demonstrated that overexpression of *MDFIC* could attenuate the bta-miR-23a-induced effects during MD.

### 3.7. MDFIC Regulate the Transcription Activity of MEF2C

**Figure 7:**
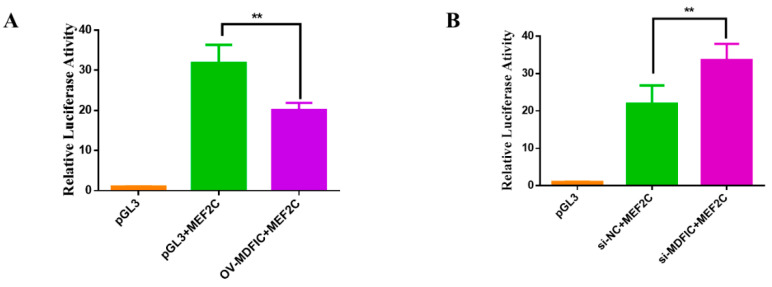
*MDFIC* regulate the transcription activity of *MEF2C*. (**A**,**B**) Luciferase assay was conducted by co-transfecting *MEF2C* promoter region and OV-MDFIC or si-MDFIC with TK-Renilla plasmid. Results are representative of the mean (± SD) of three independent analyses. ** *p* < 0.01.

To understand the relationship between *MDFIC* and *MyoG*, we cloned the promoter region of *MEF2C*, a known target gene of *MyoG* [32], into pGL3-Basic vector. The *MEF2C* promoter containing vector was co-transfected with OV-MDFIC or si-MDFIC with TK-Renilla plasmid, and the results showed that OV-MDFIC reduced the LR activity while si-MDFIC increased the LR activity (Figure 7A,B). These findings suggest that *MDFIC* may function through the interaction between *MyoG* transcription factor and *MEF2C* the promoter.

## 4. Discussion

**Figure 8:**
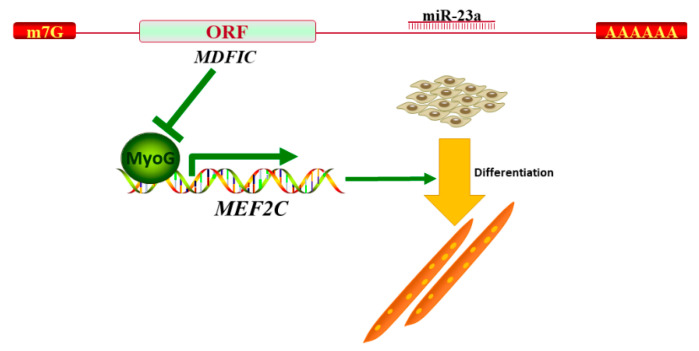
Model of miR-23a-mediated regulatory network during myoblast differentiation.

In the present work, we reveal a role for bta-miR-23a during MD of ^PDGFRα−^ bPCs. Our results indicated that bta-miR-23a controlled myoblast differentiation via targeting *MDFIC*. In addition, MDFIC regulated the transcription activity of *MEF2C* by modulating *MyoG* expression (Figure 8).

It has been demonstrated that miR-23a can modulate gene expression at the post-transcriptional level, and is involved in a broad scope of cellular processes, including cell proliferation, apoptosis, differentiation and metabolism [33,34,35]. Notably, miR-23a is responsible for the differentiation of stem cells. miR-23a is highly upregulated in mouse embryonic stem cells (mESC) to suppress differentiation toward the endoderm and ectoderm lineages, and is downregulated during the differentiation state [36]. miR-23a also maintains the balance between adipogenesis and osteogenesis in bone marrow mesenchymal stem cells [37], and plays a key factor in osteogenesis by targeting *RUNX2* and *TRPS1* [38,39]. In addition, miR-23a contributes to the early phases of neural differentiation by targeting *cyclin D1* and *Musashi1* [40,41]. Our previous works have demonstrated that bta-miR-23a can regulate the early commitment of intramuscular adipogenic differentiation by targeting *ZNF423* [30]. Moreover, previous studies have presented evidence that miR-23a plays crucial roles in muscle development and performance. Delphinidin intake can induce miR-23a expression to attenuate disuse muscle atrophy [42]. Besides, miR-23a is involved in cardiac hypertrophy via activation of muscle-specific ring finger protein 1 [43]. Furthermore, the overexpression of miR-23a and -23b have been found in the initial stages of C_2_C_12_ differentiation, and thus promote MD through suppressing TrxR1 expression [44]. However, in one study, MD can be inhibited by miR-23a via downregulation of fast MHC isoforms using mice model and mice derived cells [45]. In this study, our results indicated that bta-miR-23a could promote MD by suppressing *MDFIC*, which acted as an inhibitor of MD. The reason for this inconsistency may be explained by the different materials or the different differentiation stages focused. Here, the animal material was bovine fetus and its primary ^PDGFRα−^ bPCs. Therefore, we proposed a new mechanism for the roles of bta-miR-23a during MD.

The inhibitor of MyoD family (I-mfa) is a transcription factor encoded by MDFI, which suppresses the transactivation activities of MyoD family and inhibits skeletal myogenesis [27]. In this research, interfering MDFIC increased the transcriptional and translational levels of MyoG and MyHCs, thereby promoting the MD of ^PDGFRα−^ bPCs. Meanwhile, our rescue experiments further verified that MDFIC overexpression could reverse the inducive effects of miR-23a during MD. Some studies have suggested that I-mfa domain can target a group of bHLH proteins and repress Wnt signal transduction [46]. Moreover, I-mfa has been reported to suppress myogenesis by inhibiting lymphoid enhancer factor-1/T cell factor (LEF-1/TCF), and such inhibition can be alleviated by canonical Wnt signaling through elevating β-catenin levels [47]. Despite this, *MDFIC* and *MDFI* are belonging to the same family, and the effects of *MDFIC* on MD has not yet been experimentally clarified. Here, we provided a new insight to understand that *MEF2C* might be a key gene for the interaction between *MDFIC* and *MyoG* during MD. Taken together, all these experimental results support a conclusion that *MDFIC* is involved in myogenesis.

In conclusion, our study reveals that bta-miR-23a enhances the MD of ^PDGFRα−^ bPCs by directly targeting *MDFIC*. In addition, *MDFIC* can regulate *MEF2C* transcription activity by regulating *MyoG*. These data expand our mechanistic understanding of myogenesis and muscle development in which miRNAs play an important role.

## Figures and Tables

**Table 1:** Sequences designed for real-time PCR.

Name	Primer Sequence (5’–3’)	Annealing Temperature	Accession Number
Myogenin *(MyoG)*	F: CAAATCCACTCCCTGAAA	60 °C	NM_001111325.1
	R: GCATAGGAAGAGATGAACA		
Myosin heavy chain 1 *(MyHC1)*	F: GGGAAACTGGCTTCTGCTGAT	60 °C	NM_174117.1
	R: TGGGTTGGTGGTGATTAGGAG		
Myosin heavy chain 2 *(MyHC2)*	F: GTCAAAGGGACTATCCAGAGCAG	60 °C	NM_001166227.1
	R: AGAAGAGGCCCGAGTAGGTGT		
Myosin heavy chain 4 *(MyHC4)*	F: CTCCTAATCACCACCAACCCATA	60 °C	XM_002695806.5
	R: TGTCAGCAACTTCAGTGCCATC		
Myosin heavy chain 7 *(MyHC7)*	F: AAGACAGTGACCGTGAAGGAGG	60 °C	NM_174727.1
	R: GGTTGATGGTGACGCAGAAGA		
MyoD family inhibitor domain containing *(MDFIC)*	F: TGAGGAGGAAATAAGCAAGATAA	60 °C	NM_001101102.1
	R: CAGGATACAGTGGACACAGCAGT		
bta-miR-23a	F: ATCACATTGCCAGGGATTTCC	65 °C	NR_031347.1
18s	F: GTAACCCGTTGAACCCCATT	60 °C	NM_001025002.1
	R: CCATCCAATCGGTAGTAGCG		
U6	F: GCTTCGGCAGCACATATACTAAAAT	65 °C	NM_001075477.2

**Table 2:** Primer sequences designed for vector construction.

Name	Primer Sequence (5’–3’)	Annealing Temperature	Accession Number
Psi-CHECK-MDFIC-W	F: ACTCTCGAGACTTTTCCTTTGTTGTCTATTC	60 °C	NM_001101102.1
	R: ATAGCGGCCGCAGAGACCAAATCTGTAACTGTA		
Psi-CHECK-MDFIC-Mut	F: AAAACAAAAGTAGCAAATATTTTCCATATG	60 °C	NM_001101102.1
	R: CATATGGAAAATATTTGCTACTTTTGTTTT		
pBI-CMV3	F: TCCTTCCTAAATCTCCAGAGGATCATAATCAGCCATAC	68 °C	
	R: GATGATGATGATCGTCGACAAGCTTATCGATGC		
MDFIC	F: TGTCGACGATCATCATCATCATCATCACATGTCCGGCGCGGGCGAAG	60 °C	NM_001101102.1
	R: CTCTGGAGATTTAGGAAGGAAAACAAATCCCGCAGCACTCCATGC		
pGL3-basic	F: GGGCTCGAGATCTGCGATCTAAGTAAG	60 °C	
	R: GGGCTAGCACGCGTAAGAGC		
MEF2C	F: TCTTACGCGTGCTAGCCCTCACTTAGTATTAAAAATAGTTTGATTTTAAAAGTA GAAAGGTCATATATGAAAAACATAATAAAGTCCAGGTAAAGAAATACCTGATAG	68 °C	NM_001046113.1
	R: AGATCGCAGATCTCGAGCCCTCTATGAAGACCCAGGCTTTCCCCCCTTG		
